# Quantum Chemical Study on the Antioxidation Mechanism of Piceatannol and Isorhapontigenin toward Hydroxyl and Hydroperoxyl Radicals

**DOI:** 10.1371/journal.pone.0133259

**Published:** 2015-07-15

**Authors:** Yang Lu, AiHua Wang, Peng Shi, Hui Zhang, ZeSheng Li

**Affiliations:** 1 College of Material Science and Engineering, Harbin University of Science and Technology, Harbin, 150080, People’s Republic of China; 2 College of Chemical and Environmental Engineering, Harbin University of Science and Technology, Harbin, 150080, People’s Republic of China; 3 Key Laboratory of Cluster Science of Ministry of Education & School of Chemistry, Beijing Institute of Technology, Beijing, 100081, People’s Republic of China; University of Calgary, CANADA

## Abstract

A systematic study of the antioxidation mechanisms behind hydroxyl (•OH) and hydroperoxyl (•OOH) radical scavenging activity of piceatannol (PIC) and isorhapontigenin (ISO) was carried out using density functional theory (DFT) method. Two reaction mechanisms, abstraction (ABS) and radical adduct formation (RAF), were discussed. A total of 24 reaction pathways of scavenging •OH and •OOH with PIC and ISO were investigated in the gas phase and solution. The thermodynamic and kinetic properties of all pathways were calculated. Based on these results, we evaluated the antioxidant activity of every active site of PIC and ISO and compared the abilities of PIC and ISO to scavenge radicals. According to our results, PIC and ISO may act as effective •OH and •OOH scavengers in organism. A4-hydroxyl group is a very important active site for PIC and ISO to scavenge radicals. The introducing of -OH or -OCH_3_ group to the ortho-position of A4-hydroxyl group would increase its antioxidant activity. Meanwhile, the conformational effect was researched, the results suggest that the presence and pattern of intramolecular hydrogen bond (IHB) are considerable in determining the antioxidant activity of PIC and ISO.

## Introduction

Piceatannol (4’,5’,3,5-tetrahydroxystilbene), a natural stilbene, can be found in various plant species, such as grape [1], peanut [2], vaccinium berries [3], euphorbia lagascae [4], etc. PIC is also an analogue of resveratrol (4’,3,5-trihydroxystilbene), and can be produced from resveratrol by the action of cytochrome P450 enzyme CYP1B1 in vivo [5]. As we know resveratrol (RES) is a well known health-promoting active component with a wide variety of desirable biological activities, especially its antioxidative activity in biological systems [6,7]. The antioxidant activity of RES and other polyhydroxylated stilbenes is closely related to the location and number of hydroxyl (-OH) group which can scavenge harmful free radicals produced in vivo [8,9]. However, PIC has been pronounced to be a stronger antioxidant than RES owing to its extra-OH group [10]. Hence PIC is better than RES in increasing the lifespan of yeast cells by stimulating the activity of SIRT1 [11]. Now, PIC is getting more extensive attention because of its abirritation to age-related diseases, such as anti-inflammatory, anticarcinogenic, antiviral, antioxidative, neuroprotective and estrogenic properties [12–18].

Isorhapontigenin (3,4’,5-trihydroxy-3’-methoxy-stilbene), which is isolated from Belamcanda chinensis, is also a derivative of stilbene, and possesses a similar chemical structure with PIC. Red wine and grapes are main sources of PIC in the human diet, and recently ISO was also identified from wine grapes [19]. Generally speaking, the compounds with similar structures would have similar biological activities. Some recent studies have proved that ISO also possesses antioxidative activity with the evidence of inhibiting oxidation of human low-density lipoprotein (LDL) and other pro-oxidant system in vitro [20,21]. Fang et al. reported that ISO could inhibit the respiratory burst of PMA-activated rat neutrophils by scavenging oxygen free radicals [22]. Liu et al. showed that ISO attenuates the proliferation of bovine aortic smooth muscle cells which are induced by oxidized LDL, by blocking the generation of reactive oxygen species (ROS) and activation of the ERKs pathway [23].

Regarding the cause of aging, one of the prevalent theories is the free radical theory. In 1956, Harman proposed the concept that free radicals play an important role in the aging process of biological systems [24]. In light of his theory, the continual generation of free radicals in the process of cellular metabolism would result in the accumulation of oxidative damage to proteins, lipids and DNA, which is the primary causal factor of the aging process. Then in 1969, McCord and Fridovich discovered the superoxide dismutase (SOD) and indicated that it can disintegrate the generation of ROS from the mitochondrial respiratory chain [25]. This provided convincing evidence about the importance of free radicals in living body. Recently, it was discovered that ROS lead to the accumulation of oxidative damage to cellular constituents. This gives rise to a new development of the oxidative stress theory of aging, which holds that the increase in ROS accompanying aging can cause the functional alterations, pathological conditions, and even death [26].

ROS are certain radicals derived from oxygen. As the byproducts of normal metabolism, they represent the most important class of radicals in biological systems. ROS can cause the oxidative decomposition of cellular membranes by lipid peroxidation, and induce the DNA damage [27]. In addition, ROS are responsible for the apoptosis of cells [28]. In order to counteract the excess ROS and then avoid oxidative damage, natural antioxidant as a kind of phytoalexin, can also protect the human body from the damage of free radicals and prevent the advancement of certain chronic diseases related to oxidative stress. PIC was found to have inhibitory effect on the toxic action to neutrophils and oxidative damage to tissues, by scavenging free radicals produced by neutrophils [29].

Besides experimental studies, some theoretical research on the antioxidant activity of PIC have been conducted. Recently, Rossi et al. solved the crystal structure of PIC, and based on the structure they pointed out that the strong antioxidant activity of this polyphenol is due to the formation of the extensive hydrogen bond by its hydroxyl groups which facilitates the transfer of the hydrogen atom. They also compared the ability of RES and PIC to scavenge free radicals, and proved that PIC is a more efficient •OH and •OOH scavenger than RES [30]. Rossi et al. performed a theoretical investigation on the structural features of PIC and the effect of water using DFT method and COSMO solvation model [30]. Mikulski et al. first completed a theoretical research of the electronic structural feature of PIC using MP2 and DFT method [31]. Perez-Gonzalea et al. investigated the free radical scavenging activity of a series of polyphenols. By comparing their activity indexes and bond dissociation energy (BDE), they found that PIC has a smaller BDE, which suggested that it is a good antioxidant via H transfer [32]. Cordova-Gomez et al. studied the ability of RES and PIC anions to scavenge •OOH in water and pentyl ethanoate solution using DFT method. They indicated that PIC is a better hydroperoxyl radical scavenger than RES, regardless of the polarity of the environment [33]. Above studies provide us some knowledge on the microscopic mechanisms of the antioxidation reactions of PIC. However the theoretical research of the antioxidant reaction of ISO has not been reported. Therefore, we proceed a quantum chemical study on the activity of ISO scavenging radical for the first time. The main objective of this study is to establish a qualitative and quantitative relationship among the thermodynamic, kinetic and structural properties for PIC and ISO. By comparing the obtained barrier height of each pathway, we found out the effect of different substituent group on the ability of PIC and ISO scavenging radicals. Hope this research can provide some theoretical basis for the subsequent drug design involving the stilbenes antioxidation.

Hydroxyl and hydroperoxyl radical are the main ROS generated in vivo. Particularly to •OH, it is the most active ROS with a very short half-life of approx 10^−9^s in vivo [34]. In contrast, •OOH is the relatively slow-reacting species with half-life of the order of seconds [35], and be capable of diffusing to the remote cellular locations [36].

To research the antioxidant activity of PIC and ISO, we conducted a systematic study on the reaction mechanisms behind the •OH and •OOH radical scavenging activities of PIC and ISO, using DFT method. Two reaction mechanisms, ABS and RAF, were investigated. In addition, we obtained the thermodynamic and kinetic properties of all pathways in water to investigate the solvation effect on reactions.

## Computational Methods

All quantum chemical calculations were performed using the GAUSSIAN 09 computational package [37]. Unrestricted calculations were used for open shell systems. Geometry optimization and frequency calculation of all stationary points (reactants, complexes, transition states and products) were carried out at the M05-2X level with the 6–311++G(d,p) basis set. All of the reactants, complexes and products have only real frequencies; while all of the transition states (TS) present a single imaginary frequency which corresponds to the expected vibrational mode. The M05-2X function is a new DFT method that was specifically developed for kinetics calculation by Truhlar group [38]. So far, this functional yields satisfactory overall performance for the calculations of thermochemistry and thermochemical kinetics in organic, biological systems involving free radical reaction [39–41].

Solvation effect was introduced using SMD Continuum Solvation Model [42] which is recommended by Gaussian Manual to compute solvation energy. Because water is the major component in organism, in this work we use water as the solvent to simulate the cellular environment. The solvation effect was assessed by the single point calculations on the optimized geometries of the gas phase, with SMD model at the M05-2X/6-311++G(d,p) level of theory.

Relative Gibbs energies in solution were computed using thermodynamic cycles and Hess law which explicitly include solvation energies. For example, the thermodynamic cycle for the addition reaction of •OH with PIC is as follow:

$$\begin{array}{l} {\text{PIC}}_{\text{gas}}+\cdot {\text{OH}}_{\text{gas}}\overset{\;{\Delta \text{G}}_{\text{gas}}\;}{\to }\cdot {\left(\text{PIC-OH}\right)}_{\text{gas}}\\ {↑}_{\;\text{-}{\Delta \text{G}}_{\text{s}}(\text{PIC})}\;{↑}_{\;\text{-}{\Delta \text{G}}_{\text{s}}(\cdot \text{OH})}\;{↑}_{\;{\Delta \text{G}}_{\text{s}}\cdot (\text{PIC-OH})}\\ {\text{PIC}}_{\text{sol}}+\cdot {\text{OH}}_{\text{sol}}\;\overset{\;\Delta {\text{G}}_{\text{sol}}\;}{\to }\cdot {\left(\text{PIC-OH}\right)}_{\text{sol}} \end{array}$$

Using this strategy the Gibbs energy of reaction in solution (ΔG_sol_) can be obtained as the sum of the Gibbs energy of reaction in the gas phase (ΔG_gas_) and the difference in solvation energies (ΔΔG_s_):

$$\Delta {\text{G}}_{\text{sol}}=\Delta {\text{G}}_{\text{gas}}+\Delta \Delta {\text{G}}_{\text{s}}$$ (1)

where ΔΔG_s_ is calculated as:

$$\Delta \Delta {\text{G}}_{\text{s}}=\Delta {\text{G}}_{\text{s}}(\cdot \text{PIC-OH})\;\text{-}\;\Delta {\text{G}}_{\text{s}}(\text{PIC})\;\text{-}\;\Delta {\text{G}}_{\text{s}}(\cdot \text{OH})$$ (2)

where ΔG_s_ represents the solvation energy. In all cases, the reference state is 1M. The solvent cage effect has been included with the corrections proposed by Okuno [43], which takes into account the free volume theory. These corrections are in good agreement with those independently obtained by Ardura et al. [44]. In this work the expression used to correct the Gibbs energy is:

$${\Delta \text{G}}_{\text{sol}}^{\text{FV}}≅{\Delta \text{G}}_{\text{sol}}^{\text{0}}\;\text{-}\;\text{RT}\left\{\ln\left[n\;10^{\left(2n-2\right)}\right]\;\text{-}\;(n\;\text{-}\;1)\right\}$$ (3)

where *n* represents the molecularity of the reaction. According to the Expression 3, the solvent cage effect causes a decrease of 10.63 kJ/mol in ΔG for a bimolecular reaction at 298.15 K [43].

## Results and Discussion

It has been proved by experiments that PIC and ISO have remarkable activity of scavenging radicals. To clarify the mechanisms behind their scavenging activities, we researched the reactions of PIC and ISO with two classic ROS (•OH and •OOH) produced in organism.

**Fig 1:**
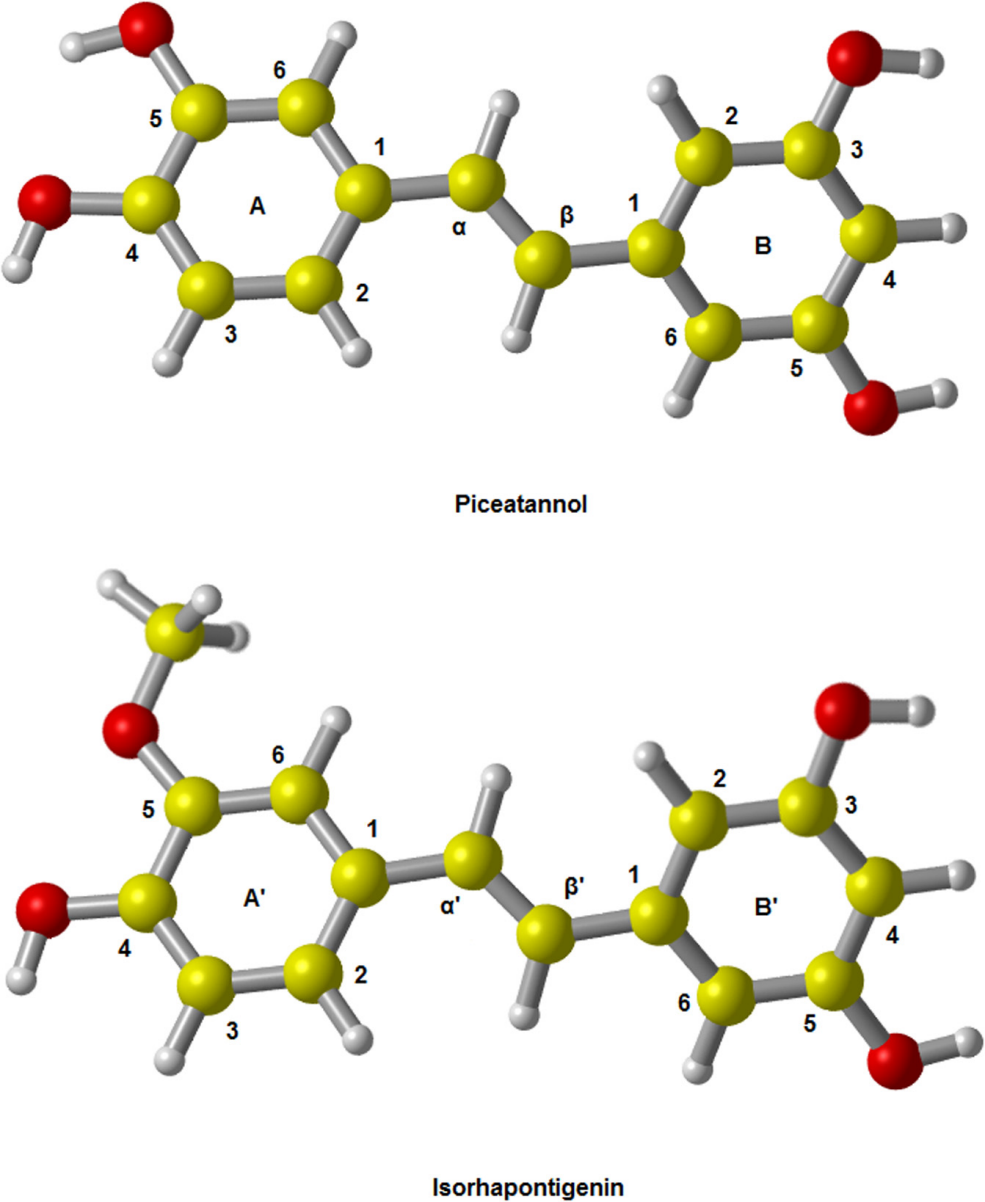
The optimized geometries of PIC and ISO in the gas phase. The results show that both of PIC and ISO have an approximately planar structure. The dihedral angle between two benzene rings of PIC is about 179.95°, which is very consistent with the experimental result of 179.23°; the corresponding dihedral angel of ISO is -179.90°.

The optimized structures of PIC and ISO with atom labels are shown in Fig 1. By our calculation with the method of M05-2X/6-311++G(d,p), the dihedral angle between two benzene rings of PIC is 179.95°, which is similar with the value of 179.50°calculated by MP2/6-311G(d,p) method [31]. The theoretical results are in agreement well with the corresponding experimental value of 179.23° [30]. For each kind of free radical, we considered four abstraction reaction pathways (from the site A4, A5, B3 and B5 in Fig 1) by ABS mechanism as well as two addition reaction pathways (adding to the site α and β) by RAF mechanism. At the same time, the solvent effect was also taken into account.

### Hydroxyl Radical (•OH)

•OH abstracts the H atom from the hydroxyl group of PIC or ISO, followed by forming a water molecule and a corresponding radical; while at the A5 site of ISO, •OH abstracts CH_3_ from the methoxyl group. In general, the-OH group of different site possesses different activity of scavenging radicals. So we defined eight ABS pathways: A4, A5, B3, B5 for PIC and A'4, A'5, B'3, B'5 for ISO. The ABS reactions can be expressed as follow:

$$\text{PIC}+\cdot \text{OH}\to \cdot \text{PIC}(\text{-}H)+{\text{H}}_{2}\text{O}$$ (4)

$$\text{ISO}+\cdot \text{OH}\to \cdot \text{ISO}(\text{-}H)+{\text{H}}_{2}\text{O}$$ (5)

$$\text{ISO}+\cdot \text{OH}\to \cdot \text{ISO}(\text{-}{\text{CH}}_{3})+{\text{CH}}_{3}\text{OH}$$ (6)

**Fig 2:**
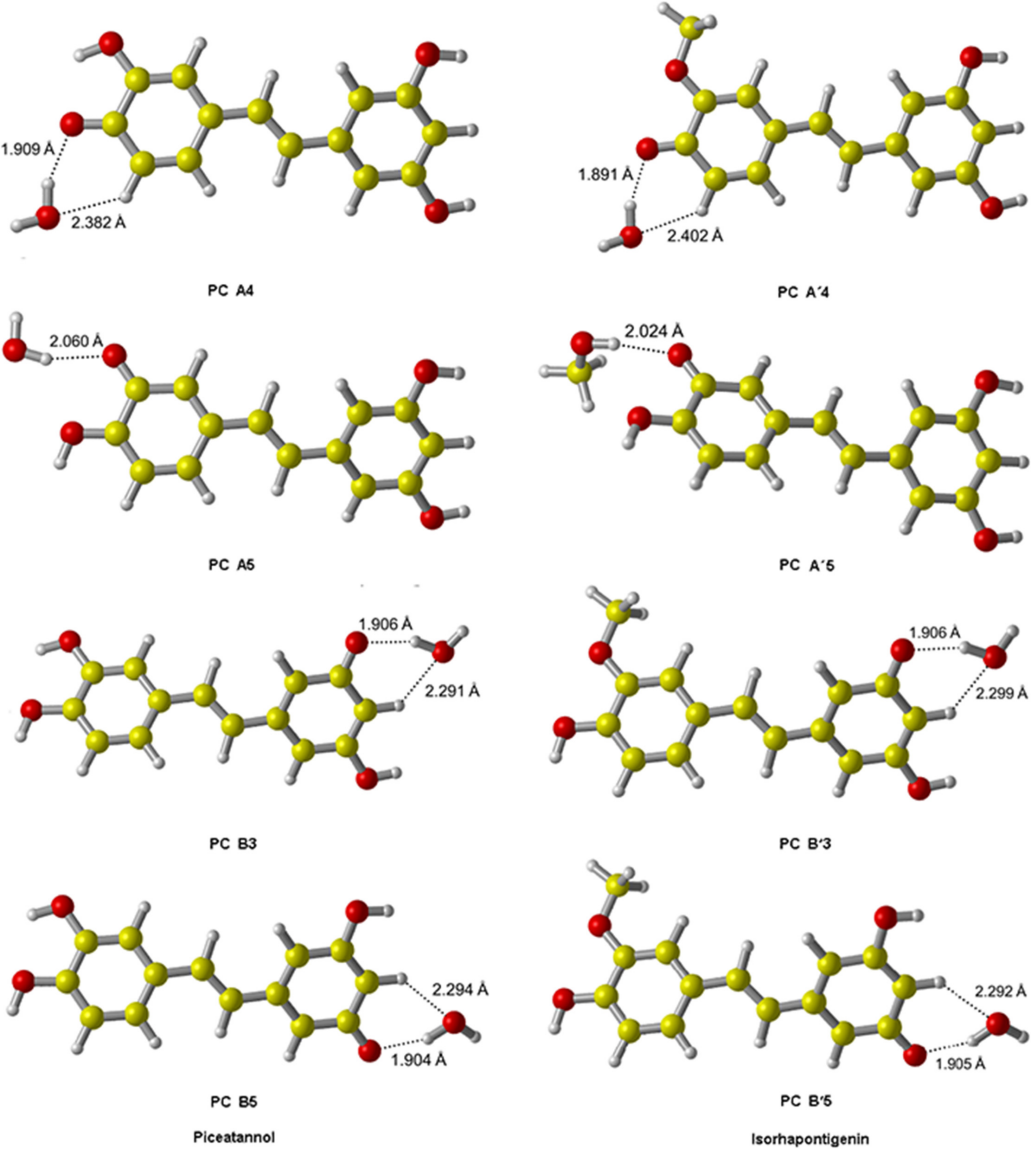
The optimized geometries of the product complexes of PIC and ISO from ABS reactions initiated by •OH. In these structures, the water molecule forms a hydrogen bond with the PIC/ISO semiquinone radical.

The main products after the H atom has been abstracted are semiquinone radicals which are stable as that the unpaired electron can delocalize through both aromatic rings. The optimized structures of all product complexes (PC) are shown in Fig 2.

For completely separated reactants and products, the reaction Gibbs energies (ΔG_gas_) and reaction enthalpies (ΔH_gas_) of ABS reactions are presented in Tables 1 and 2 respectively. It can be seen, for both of PIC and ISO, all ABS pathways are exothermic and thermodynamically favored. Among them, the most exergonic pathway is from the same position, A4 and A'4. Therefore the semiquinone radicals produced from A4 and A'4 pathway are expected to be the major products of ABS reactions. This is because the resonance effect between two aromatic rings can donate the electron pairs which can be transmitted via the stilbene bridge, and this type of conjugation in electron transfer process has been reported previously [45].

**Table 1:** The reaction Gibbs energies for reactions of PIC and ISO with •OH in the gas phase and water (in kJ/mol).

	PIC+•OH			ISO+•OH	
site	ΔG _gas_	ΔG _sol_	site	ΔG_gas_	ΔG_sol_
**A4**	-181.55	-196.19	**A'4**	-162.75	-190.32
**A5**	-121.18	-164.78	**A'5**	-133.75	-161.70
**B3**	-40.23	-42.61	**B'3**	-39.94	-42.32
**B5**	-38.17	-40.01	**B'5**	-40.86	-43.29
**α**	-105.84	-104.00	**α'**	-108.12	-108.04
**β**	-105.03	-108.12	**β'**	-108.93	-111.07

**Table 2:** The reaction enthalpies at 298K for reactions of PIC and ISO with •OH in the gas phase and water (in kJ/mol).

	PIC+•OH			ISO+•OH	
site	ΔH_gas_	ΔH_sol_	site	ΔH_gas_	ΔH_sol_
**A4**	-171.31	-176.54	**A'4**	-156.76	-174.16
**A5**	-118.10	-150.86	**A'5**	-122.39	-139.56
**B3**	-36.63	-129.55	**B'3**	-36.43	-132.32
**B5**	-36.47	-130.70	**B'5**	-36.92	-131.56
**α**	-150.60	-141.15	**α'**	-151.34	-146.56
**β**	-150.45	-147.02	**β'**	-151.96	-147.29

**Fig 3:**
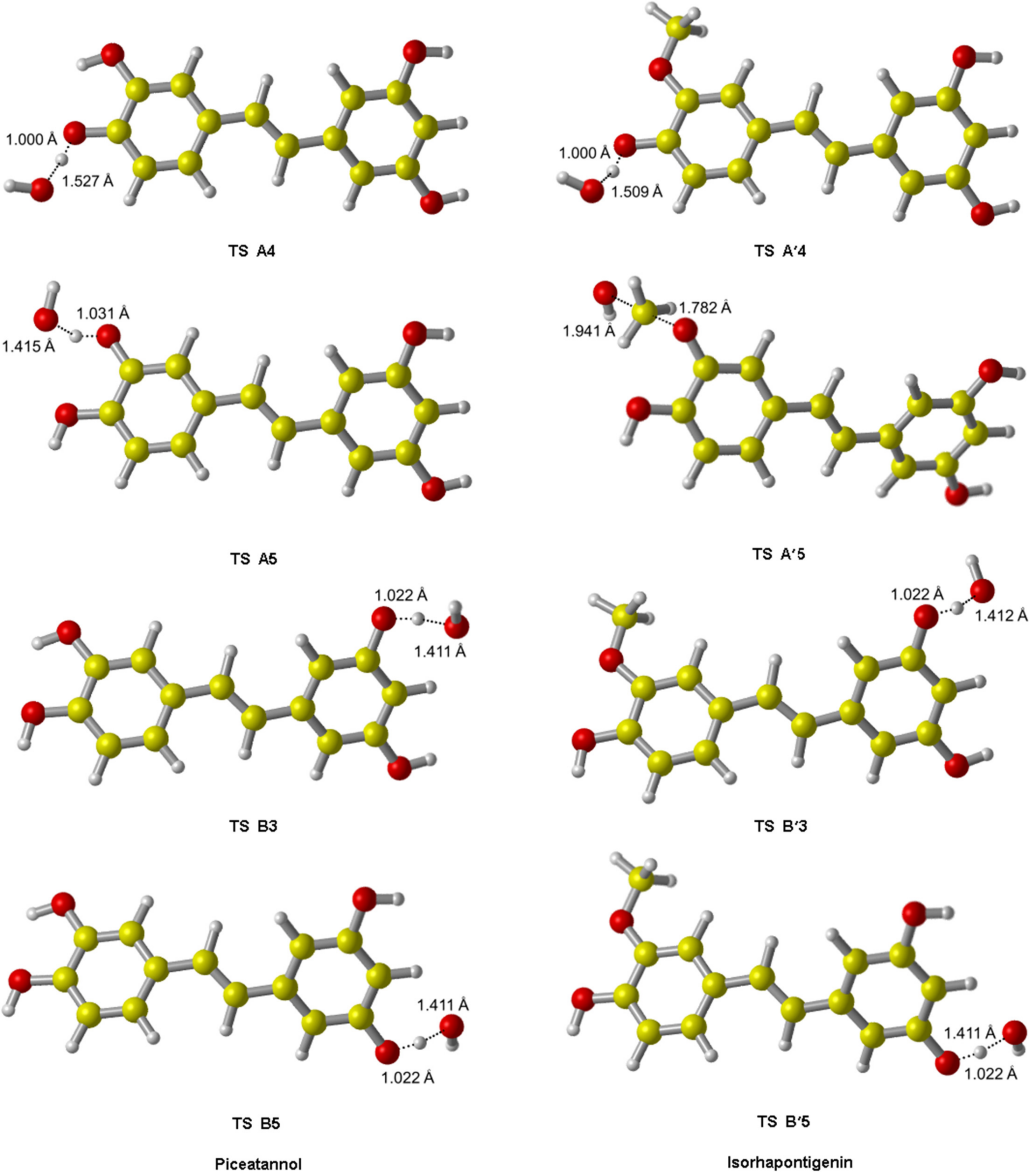
The transition state geometries of PIC and ISO from ABS reactions initiated by •OH. The elongations of the breaking bonds are smaller than those of the forming bonds, indicating these TS are all reactant-like, i.e. these reactions are all exothermic in light of the Hammond’s postulate. These agree with our calculated results in Table 2.

We identified all TS of ABS reactions in the gas phase at the M05-2X/6-311++G(d,p) level, their fully optimized geometries are shown in Fig 3. For the TS structures of A4, A5, B3 and B5, the lengths of O‒H bonds that need to break are increased by 4.3%, 7.1%, 6.6% and 6.6% respectively comparing to their equilibrium bond lengths in PIC reactant; the lengths of H‒O bonds that are ready to form are stretched by 59.6%, 47.9%, 47.4% and 47.4% respectively comparing to their equilibrium bond lengths in isolated H_2_O product. Thus, all the breaking bonds are much less elongated than all the forming bonds, indicating that all TS of PIC in ABS pathways are reactant-like. Similarly, all TS from ABS reactions of ISO are reactant-like. These are consistent with the Hammond’s postulate [46] for exothermic reaction.

**Fig 4:**
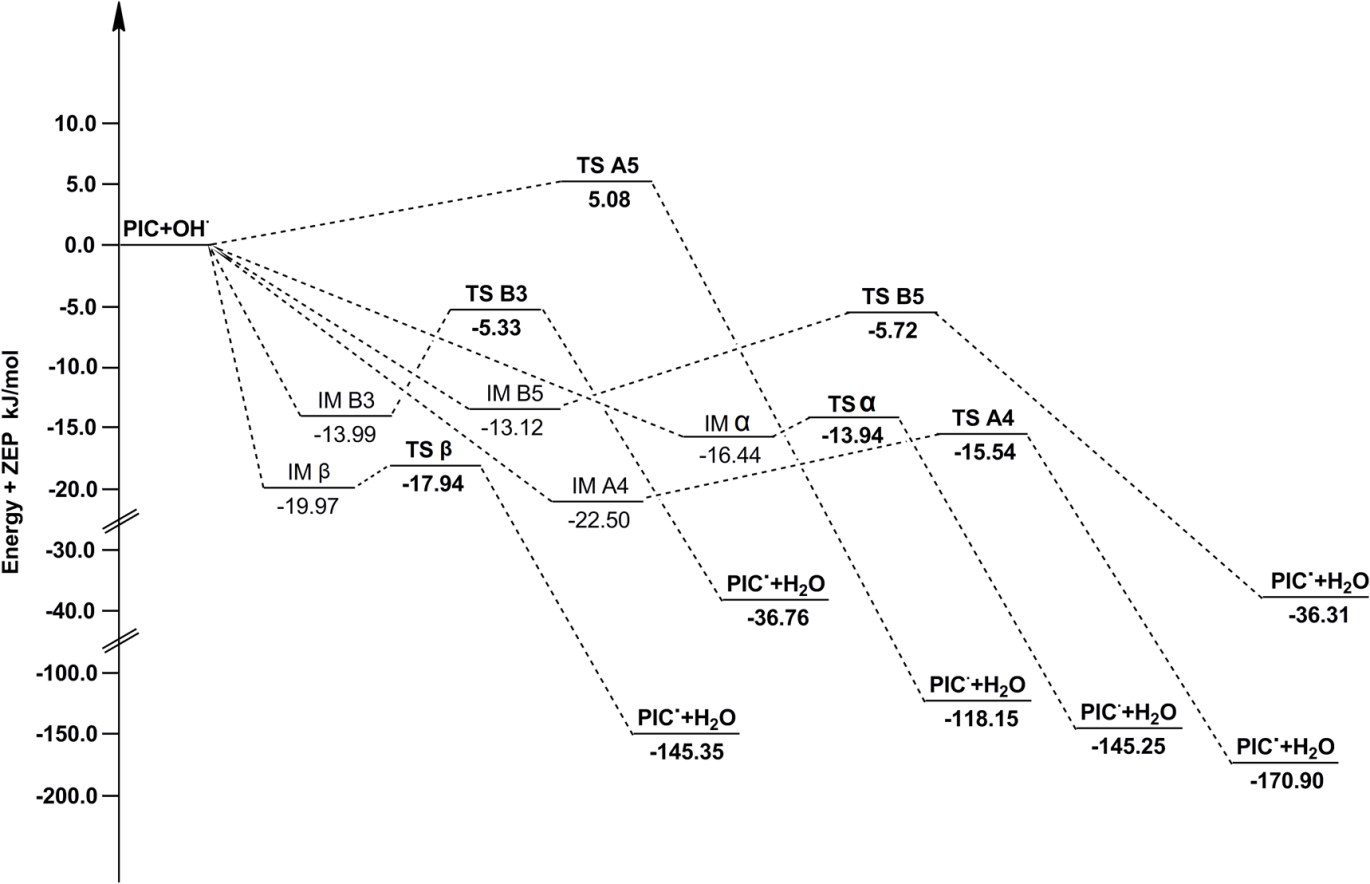
The potential energy surfaces of the reactions of PIC with •OH in the gas phase. The relative energies (in kJ/mol) were calculated at the M05-2X/6-311++G(d,p) + ZPE level. To facilitate comparison, the energy of the reactants are set to zero.

**Fig 5:**
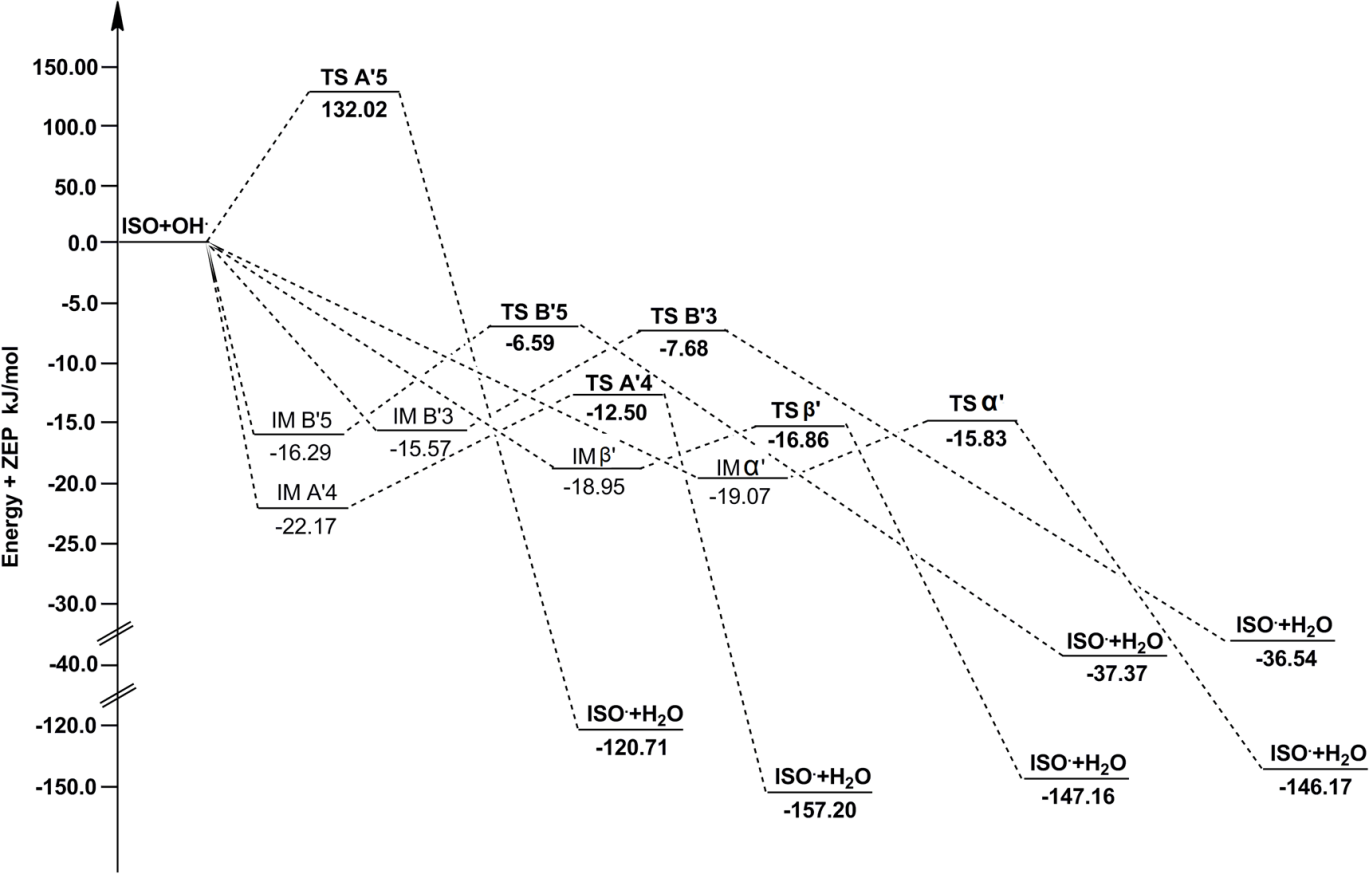
The potential energy surfaces of the reactions of ISO with •OH in the gas phase. The relative energies (in kJ/mol) were calculated at the M05-2X/6-311++G(d,p) + ZPE level. To facilitate comparison, the energy of the reactants are set to zero.

The barrier heights including zero-point energy corrections for ABS pathways are reported in Table 3. It can be found that most pathways in Table 3 have negative barrier heights, these reactions are barrierless reactions. The reactant complexes (IMA4, IMA'4, IMB3, IMB'3, IMB5, IMB'5, IMα, IMα', IMβ and IMβ') are formed in the entrance of pathway A4, A'4, B3, B'3, B5, B'5, α, α', β and β'. The energies of IM are lower than that of reactants, the relative energies (the reactant energies are set to zero) were plotted in Figs 4 and 5.

**Table 3:** The barrier heights of TS with zero-point energy corrections for reactions of PIC and ISO with •OH in the gas phase and water (in kJ/mol).

	PIC+•OH			ISO+•OH	
site	ΔE_gas_	ΔE_sol_	site	ΔE_gas_	ΔE_sol_
**A4**	-15.54	-58.86	**A'4**	-12.50	-56.45
**A5**	5.08	-30.58	**A'5**	132.02	98.28
**B3**	-5.33	-16.52	**B'3**	-7.68	-25.83
**B5**	-5.72	-16.44	**B'5**	-6.59	-25.25
**α**	-13.94	-15.64	**α'**	-15.83	-13.38
**β**	-17.94	-16.30	**β'**	-16.86	-13.52

We can also find, in the gas phase, the pathway A4 has the lowest barrier height in all ABS reactions of PIC with •OH. Combining with the thermodynamic analysis, we conclude that the pathway A4 is more thermodynamiclly and kinetically favorable than other ABS pathways, and it is the major •OH scavenging pathway of PIC by ABS mechanism. This finding could be further confirmed by our calculation of BDE. The homolytic BDE is an important factor in determination of the effectiveness of an antioxidant: the weaker this bond, the higher its antioxidant activity. The values of O‒H BDE for sites of PIC are 310 kJ/mol (A4), 363 kJ/mol (A5), 444 kJ/mol (B3) and 445 kJ/mol (B5). The O‒H BDE of A4 site is the smallest, suggesting that its antioxidant activity is the strongest. This is coincident with the calculation result of the previous research [32]. For ISO, the pathway A'4 has the lowest barrier height in all ABS reactions in the gas phase. So the pathway A'4 is the leading channel for ISO to scavenge •OH by ABS mechanism.

In addition, addition reactions may occur as follows:

PIC+·OH→·(PIC-OH) (7)

ISO+·OH→·(ISO-OH) (8)

where •OH is added to either carbon atom of >C = C< moiety (at site α/α' and β/β', corresponding to the pathway α and β for PIC, α' and β' for ISO), and an OH-adduct radical is produced. The reaction Gibbs energies (ΔG_gas_) and reaction enthalpies (ΔH_gas_) of RAF pathways were also calculated and presented in Tables 1 and 2 respectively. The results indicate that RAF pathways of PIC and ISO are all exothermic and thermodynamically favored.

**Fig 6:**
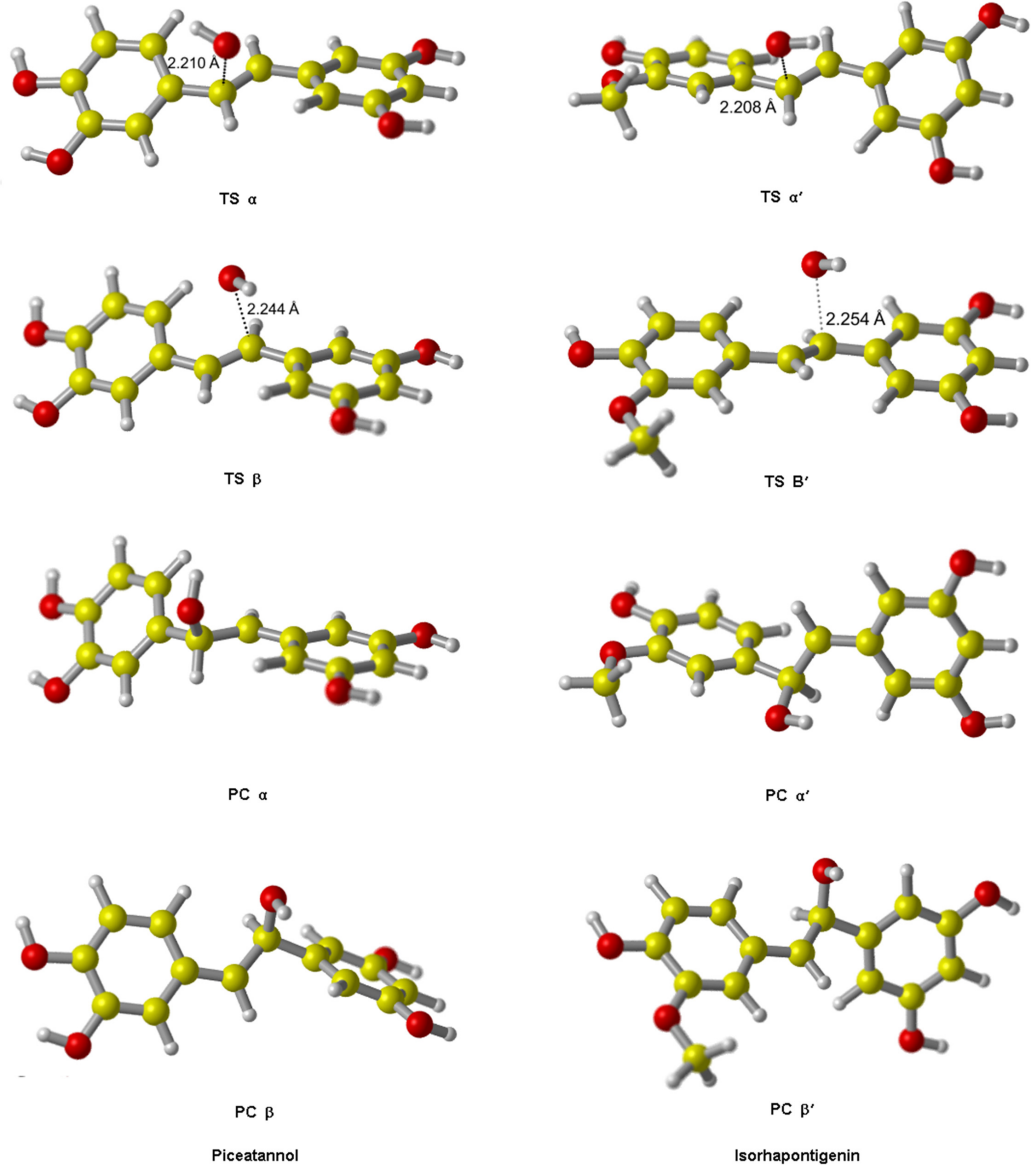
The transition states and product complexes of PIC and ISO from RAF reaction initiated by •OH. All TS have only one imaginary frequency, and in their vibrational mode, •OH moves in a direction which is perpendicular to that of the carbon atom. The H-atom attached to that carbon atom folds back slightly to accommodate the incoming of •OH.

The optimized geometries of TS and PC for addition reactions are shown in Fig 6, and the barrier heights are listed in Table 3. By comparing the value of ΔE_gas_, we estimated that the site β/β' is always more active than α/α' for PIC and ISO.

Considering the application of antioxidant scavenger in vivo environment, the reaction Gibbs energies (ΔG_sol_) and reaction enthalpies (ΔH_sol_) of all pathways in water were calculated using SMD model. The results are included in Tables 1 and 2 respectively. As same as in the gas phase, all pathways of PIC and ISO with •OH are exothermic and thermodynamically feasible in the water phase. In addition, we found that for ABS pathway, ΔG_sol_ is always smaller than ΔG_gas_, which suggests that the ABS reactions in water are more exergonic and easier than those in the gas phase. In water, the most exergonic pathways are still A4 and A'4 corresponding to the reaction systems of PIC and ISO. Thus, the thermodynamic properties of ABS reactions in water is same as in the gas phase.

The barrier heights for reactions of PIC and ISO with •OH in water were obtained and presented in Table 3. As shown in this table, the barrier heights of ABS reactions in water are significantly decreased in comparison with reactions in the gas phase. It proves that water solvent promotes the scavenging activity of PIC and ISO toward •OH. In water, the pathway A4 and A'4 respectively have the lowest barrier heights of ABS reactions just like in the gas phase. Therefore we inferred that, in the gas phase and water, A4/A'4-OH group is always the most active site of PIC/ISO by ABS mechanism, while β/β' site is always more active for RAF mechanisms.

In conclusion, A4-OH group is the dominant active site for the antioxidant reaction of PIC and ISO toward •OH. The common feature of A4-OH groups in PIC and ISO is that there is a substituent group on their ortho-position, where is a hydroxyl group in PIC and a methoxyl group in ISO. So the existence of the adjacent substituent may have a promoting effect on the antioxidant activity of A4-OH group. In addition, by comparing the best active site of PIC with that of ISO, we found that the barrier height of A4 is smaller than that of A'4 wherever in the gas phase or water, hence the activity of A4-OH group of PIC is stronger than that of ISO. This implies that, for the activity of-OH group scavenging •OH, the contribution of the adjacent-OH group is bigger than that of the adjacent-OCH_3_ group. It is probably because two adjacent hydroxyl groups of PIC can form a IHB which make the ABS product more stable, so A4-OH group becomes a better H atom donor [47].

To sum up, PIC and ISO are capable of scavenging •OH by two mechanisms and the most active site is A4-OH group for ABS mechanism and β carbon atom for RAF mechanism.

### Hydroperoxyl Radical (•OOH)

In order to investigate the scavenging activity of PIC and ISO toward •OOH, similar to •OH, eight ABS sites (A4p /A'4p, A5p/A'5p, B3p/B'3p and B5p/B'5p) and four RAF sites (αp/α'p and βp/β'p) were studied, and the corresponding PIC/ISO reaction pathways were denoted as A4p/A'4p, A5p/A'5p, B3p/B'3p, B5p/B'5p, αp/α'p and βp/β'p respectively, according to the following reactions:

$$\text{PIC}+\cdot \text{OOH}\to \cdot \text{PIC}(\text{-}H)+{\text{H}}_{2}{\text{O}}_{2}$$ (9)

PIC+·OOH→·(PIC-OOH) (10)

$$\text{ISO}+\cdot \text{OOH}\to \cdot \text{ISO}(\text{-}H)+{\text{H}}_{2}{\text{O}}_{2}$$ (11)

$$\text{ISO}+\cdot \text{OOH}\to \cdot \text{ISO}(\text{-}{\text{CH}}_{3})+{\text{CH}}_{3}\text{OOH}$$ (12)

ISO+·OOH→·(ISO-OOH) (13)

For the reactions initiated by •OOH, the reaction Gibbs energies and reaction enthalpies are reported in Tables 4 and 5 respectively (including in the gas phase and water).

**Table 4:** The reaction Gibbs energies for reactions of PIC and ISO with •OOH in the gas phase and water (in kJ/mol).

	PIC+•OOH			ISO+•OOH	
site	ΔG _gas_	ΔG _sol_	site	ΔG_gas_	ΔG_sol_
**A4p**	-44.59	-55.72	**A'4p**	-25.78	-49.84
**A5p**	15.78	-24.30	**A'5p**	-27.29	-52.02
**B3p**	96.74	97.87	**B'3p**	97.03	98.16
**B5p**	98.80	100.47	**B'5p**	96.10	97.19
**αp**	5.20	7.80	**α'p**	0.99	4.26
**βp**	1.11	2.66	**β'p**	-2.61	-4.78

**Table 5:** The reaction enthalpies at 298K for reactions of PIC and ISO with •OOH in the gas phase and water (in kJ/mol).

	PIC+•OOH			ISO+•OOH	
site	ΔH_gas_	ΔH_sol_	site	ΔH_gas_	ΔH_sol_
**A4p**	-38.19	-41.34	**A'4p**	-23.65	-38.95
**A5p**	15.02	-15.66	**A'5p**	-22.01	-38.94
**B3p**	96.49	5.66	**B'3p**	96.68	3.89
**B5p**	96.65	4.51	**B'5p**	96.20	3.56
**αp**	-47.09	-44.97	**α'p**	-48.25	-44.62
**βp**	-52.81	-43.06	**β'p**	-51.23	-49.26

Among the pathways of PIC in the gas phase, only A4p is thermodynamically feasible. So the semiquinone radical A4p is the main product of ABS reactions in the gas phase. In water, the ΔG_sol_ of pathway A4p and A5p are both negative as shown in Table 4, i.e. the ABS reactions in ring A are thermodynamically feasible. In contrast, the pathways in ring B are not thermodynamically viable. These are in accord with the previous study [33]. In addition, all RAF pathways are endergonic both in the gas phase and water, so none of addition reaction can happen spontaneously. It is possible to conclude that PIC cannot scavenge •OOH by RAF mechanism.

For ISO, pathway A'4p and A'5p are exergonic and exothermic both in the gas phase and water. As same as PIC, only the the reactions in ring A of ISO can happen spontaneously, the ring B has no contribution to scavenge •OOH. In addition, the RAF pathway β'p is thermodynamically feasible. So ISO can scavenge •OOH radical by both of ABS and RAF mechanisms.

**Fig 7:**
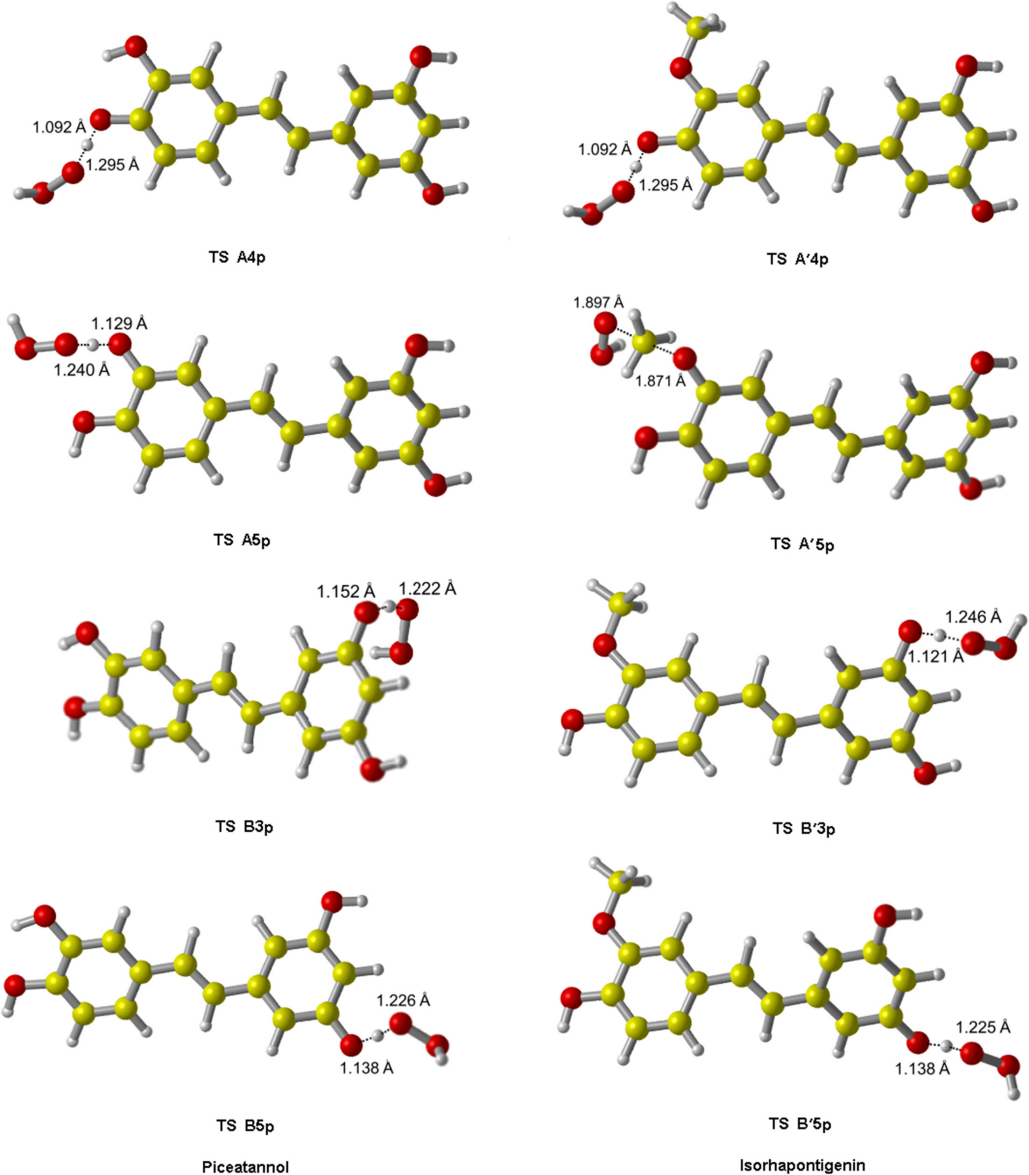
The transition state geometries of PIC and ISO from ABS reaction initiated by •OOH. For the same active site of PIC and ISO, the corresponding TS structures are very similar.

**Fig 8:**
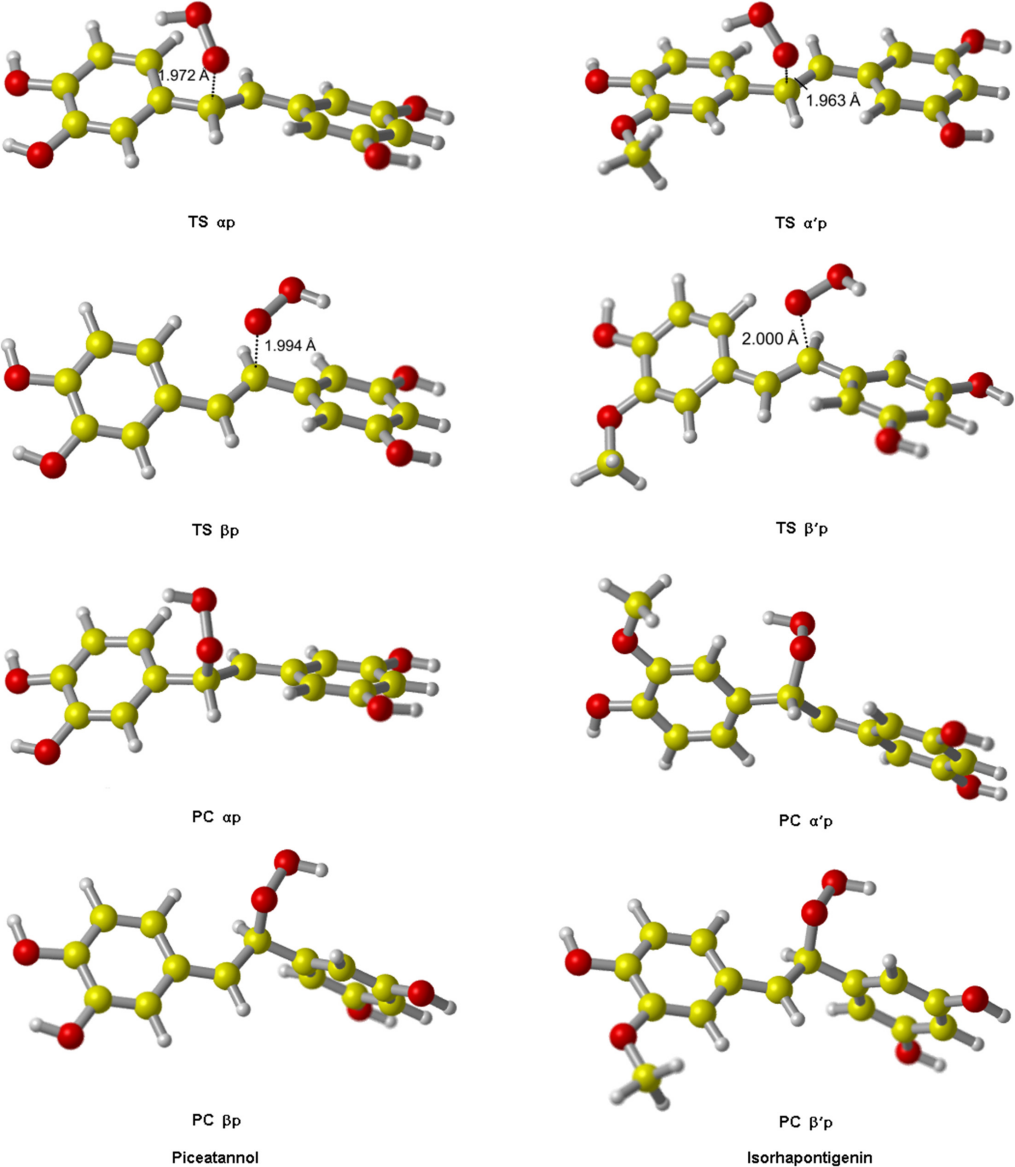
The transition states and product complexes of PIC and ISO from RAF reaction initiated by •OOH. All TS have only one imaginary frequency, and in their vibrational mode, •OOH moves in a direction which is perpendicular to that of the carbon atom. The H atom attached to that carbon atom folds back slightly to accommodate the incoming of •OOH.

All TS of ABS reactions were identified and shown in Fig 7. In each of these structures, •OOH is approximately perpendicular to the molecular plane of PIC or ISO, and orientated toward the H atom of the-OH group. All TS and PC of RAF reactions initiated by •OOH are shown in Fig 8.

**Fig 9:**
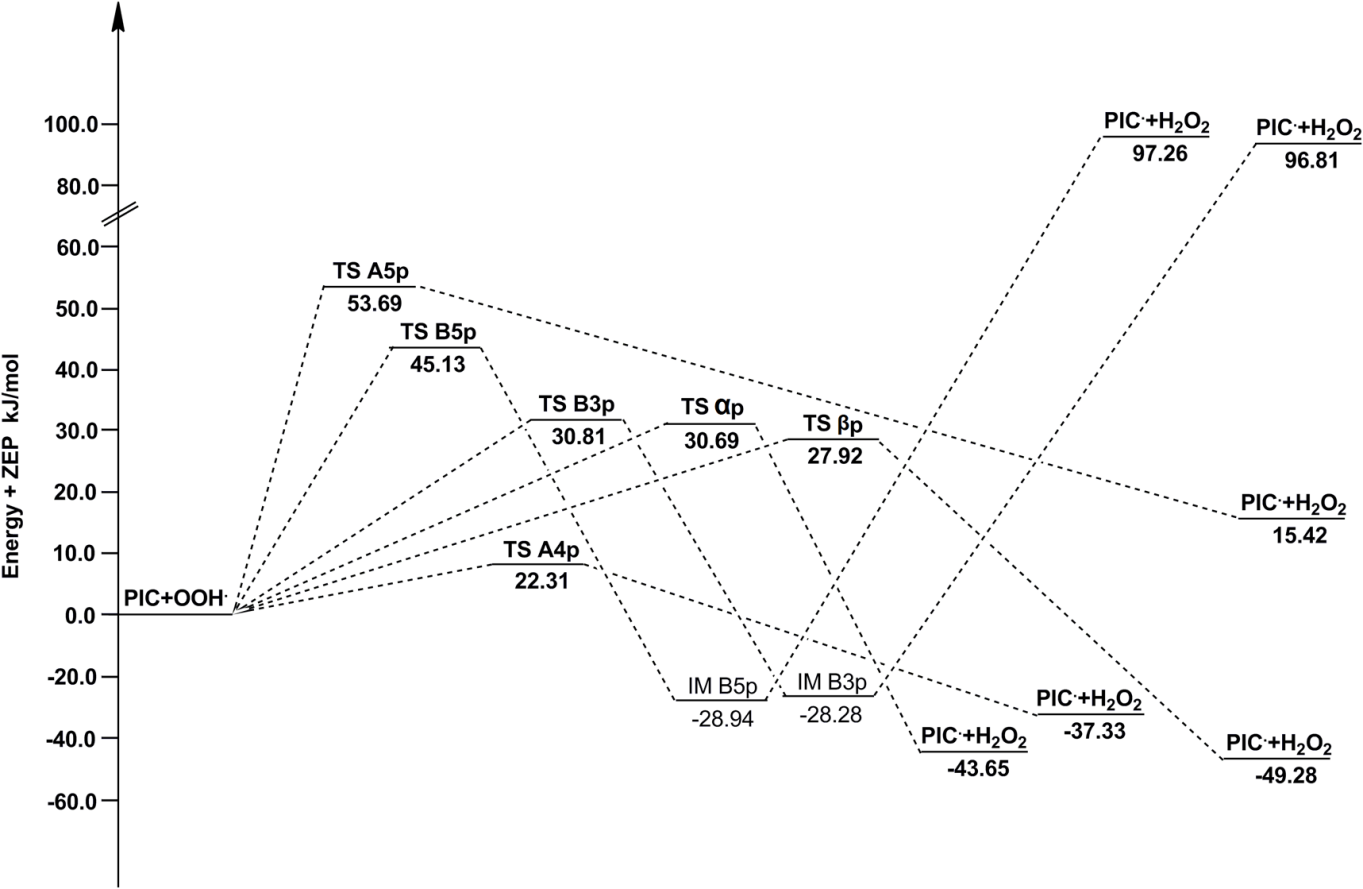
The potential energy surfaces of the reactions of PIC with •OOH in the gas phase. The relative energies (in kJ/mol) were calculated at the M05-2X/6-311++G(d,p) + ZPE level. To facilitate comparisons, the energy of the reactants are set to zero.

**Fig 10:**
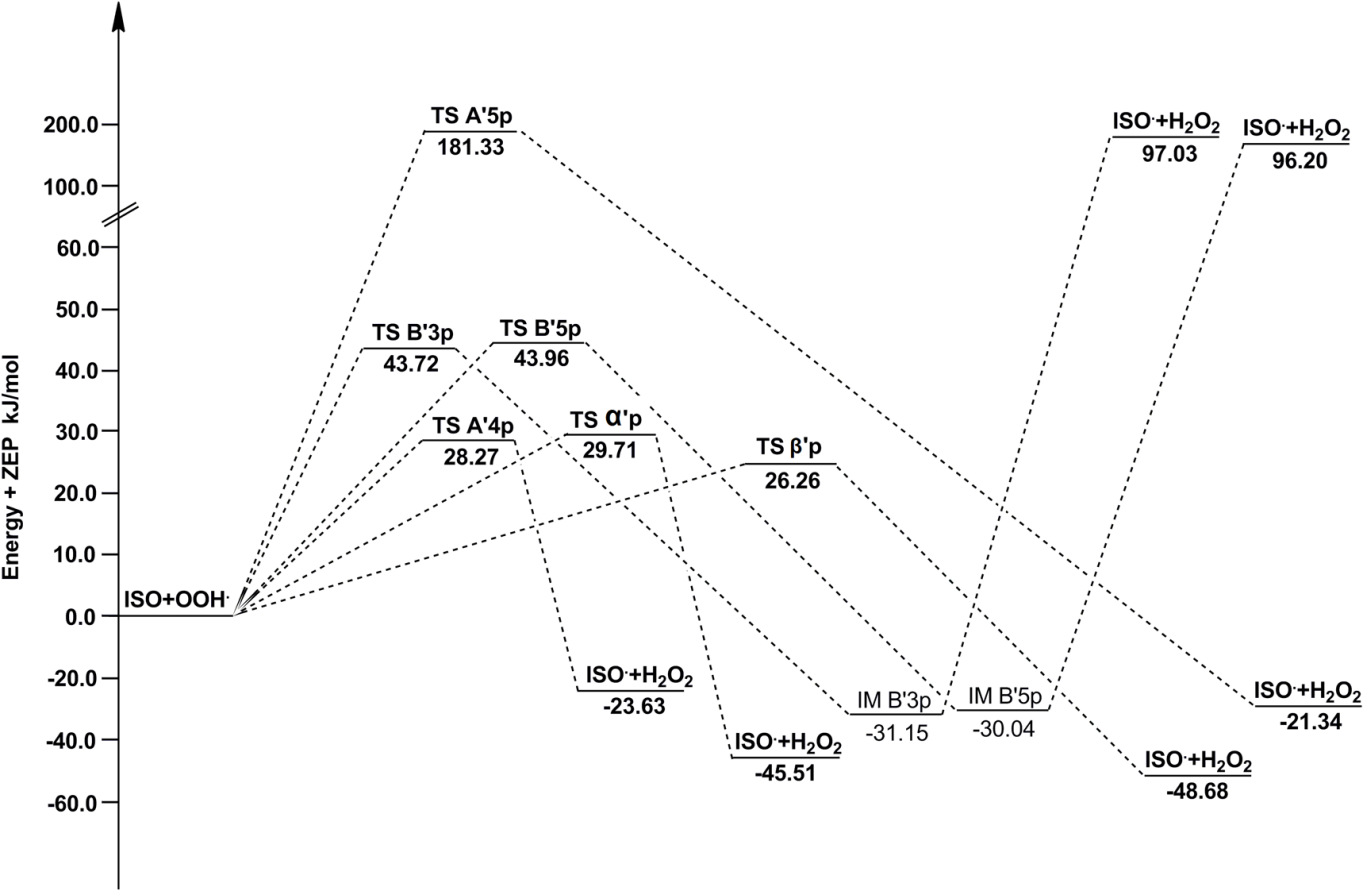
The potential energy surfaces of the reactions of ISO with •OOH in the gas phase. The relative energies (in kJ/mol) were calculated at the M05-2X/6-311++G(d,p) + ZPE level. To facilitate comparisons, the energy of the reactants are set to zero.

The barrier heights of all reactions in the gas phase and water are reported in Table 6. Potential energy diagrams are plotted in Figs 9 and 10. As shown in Table 6, for ABS reactions of PIC in the gas phase, the pathway A4p has the lowest barrier height. While in water, A5p becomes the pathway with the lowest barrier height, where the barrier height decreases from 53.69 kJ/mol (in the gas phase) to 38.14 kJ/mol. Therefore, A5p-OH group is also an important active site for PIC to scavenge •OOH in vivo. The high activities of site A4p and A5p are due to the fact that there are two ortho-OH groups in the benzene ring A, and their respective semiquinone radical products yielded by abstraction reactions are more stable due to the formation of IHB interactions [48].

**Table 6:** The barrier heights of TS with zero-point energy corrections for reactions of PIC and ISO with •OOH in the gas phase and water (in kJ/mol).

	PIC+•OOH			ISO+•OOH	
site	ΔE_gas_	ΔE_sol_	site	ΔE_gas_	ΔE_sol_
**A4p**	22.31	50.53	**A'4p**	28.27	15.70
**A5p**	53.69	30.36	**A'5p**	181.33	166.99
**B3p**	30.81	48.87	**B'3p**	43.72	56.29
**B5p**	45.13	74.63	**B'5p**	43.96	50.36
**αp**	30.69	35.32	**α'p**	29.71	33.33
**βp**	27.92	35.15	**β'p**	26.26	33.26

All above indicate that the scavenging activity of PIC toward •OOH is ruled by the hydroxyl groups in ring A.

For ABS reactions of ISO, the pathway A'4p has the lowest barrier height wherever in the gas phase or water. Along with its thermodynamic superiority, A'4p is the absolute leading channel for ISO to scavenge •OOH by ABS mechanism. The higher activity of A'4p is as the presence of the adjacent-OCH_3_ group, because its character of electron-donating group (-OCH_3_) promotes the H atom transfer of A'4 group. Moreover, unlike PIC, the barrier height of A'5p of ISO are very big. It is probably because the steric hindrance effect brought by-OCH_3_ group is bigger than that of-OH group, which results in the decrease of its activity. For the RAF reactions initiated by •OOH, the site β'p is always more active than α'p,as same as the RAF mechanism of ISO with •OH.

In the gas phase, A4p and A'4p are the best active sites for PIC and ISO respectively, and the pathway A4p has a smaller ΔE than A'4p, indicating that the ability of PIC eliminating •OOH is superior to ISO. This is consistent with the result of eliminating •OH. But in water, ISO (A'4p) possess a smallest barrier height among all PIC and ISO pathways, so it is a better scavenger toward •OOH in vivo.

On the basis of the thermodynamic and kinetic results obtained from the reactions of scavenging •OH and •OOH, we also found that both of PIC and ISO have a higher ability to scavenge •OH than •OOH. This is reasonable, since the half-time of •OOH is several orders longer than that of •OH, and the activity of •OH is higher than •OOH.

### Conformational Analysis

**Fig 11:**
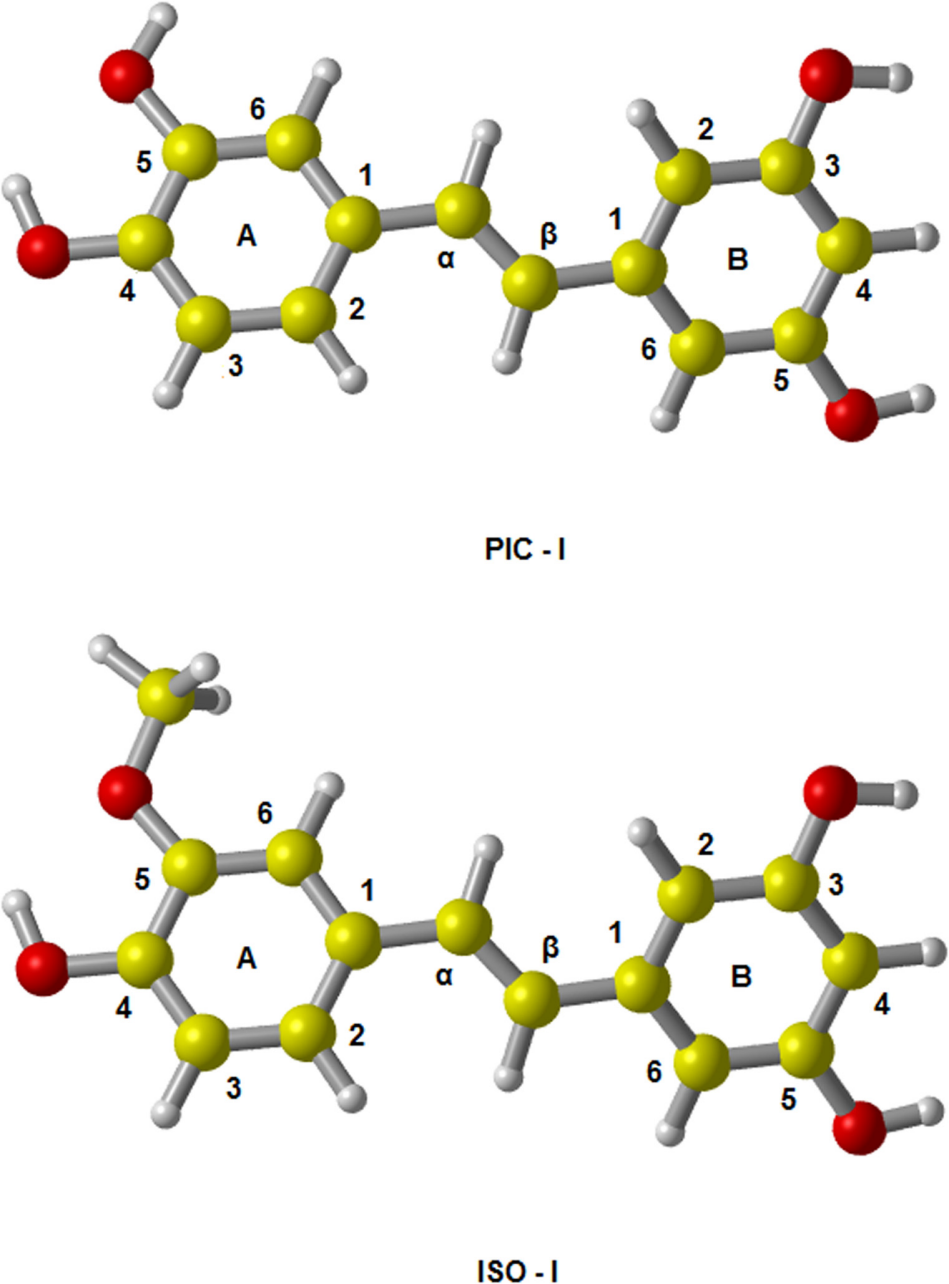
The optimized geometries of PIC-I and ISO-I in the gas phase. The dihedral angle between two benzene rings of PIC-I is 179.87°, and the dihedral angle between two benzene rings of ISO-I is -179.99°.

Conformational analysis is an important tool to investigate the antioxidant capacity of polyphenols, since the antioxidant behavior of-OH group is strongly influenced by the geometry, such as IHB. In order to clarify the probable effect of IHB to antioxidant activity of the studied compounds, we optimized the conformers of PIC and ISO which structures exist different IHB. The geometries of the conformers (PIC-I and ISO-I) with labels are shown in Fig 11. The structure features of PIC-I and ISO-I are A4-I hydroxy group form a IHB with the O atom of the adjacent A5-I hydroxy group, while the structures of ring B are not changed. So we select two active sites (A4-I and A5-I) in ring A as the target and make comparing between the same sites of PIC and ISO.

By the same method of M05-2X/6-311++G(d,p), we calculated the thermodynamic data of PIC-I and ISO-I scavenging •OH radicals (in Table 7). This table shows that the ABS reactions of PIC-I and ISO-I with •OH are all exothermic and thermodynamically favored.

**Table 7:** The reaction Gibbs energies and reaction energies at 298K for reactions of PIC-I and ISO-I with •OH in the gas phase and water (in kJ/mol).

site	PIC-I + •OH				ISO-I + •OH			
	ΔG_gas_	ΔG_sol_	ΔH_gas_	ΔH_sol_	ΔG_gas_	ΔG_sol_	ΔH_gas_	ΔH_sol_
**A4-I**	-137.56	-182.75	-133.28	-167.15	-140.80	-185.73	-135.11	-170.12
**A5-I**	-160.40	-173.91	-157.12	-160.23	-149.80	-164.24	-140.10	-145.10

As we have mentioned above, BDE is an important indicator of evaluating the activity of-OH groups in polyphenols, the relative low O‒H BED will facilitate their H-transfer ability. So we computed the O‒H BDE of PIC/ISO along with their conformers, and listed in Table 8.

**Table 8:** The bond dissociation enthalpies of O‒H bonds and the barrier heights of TS with zero-point energy corrections for reactions of PIC-I/PIC and ISO-I/ISO with •OH in the gas phase and water (in kJ/mol).

	PIC-I/PIC + •OH			ISO-I/ISO + •OH		
site	BDE	ΔE_gas_	ΔE_sol_	BDE	ΔE_gas_	ΔE_sol_
**A4-I**	347.41	2.39	-39.16	324.00	0.83	-42.91
**A5-I**	324.42	-12.87	-50.51	-	-	-
**A4**	310.30	-15.54	-58.86	324.00	-12.50	-56.45
**A5**	363.05	5.08	-30.58	-	-	-

It has been reported that IHB could stabilize the parent molecule and the radical formed after H atom has been abstracted, but the effect to the latter is more strong. Therefore the BDE of-OH group which participates the formation of IHB will be increased and the BDE of free-OH group will be decreased [49]. For the A4-I hydroxy group of PIC-I, it involves a OH^…^O hydrogen bond with the O atom of the adjacent hydroxy group; in contrast, the A4-OH group of PIC is free. So from Table 8, the O‒H BDE of A4-I is higher than that of A4, this means the activity of A4-I is decreased. What is more, the BDE of A5-I hydroxy group which is free in PIC-I is lower than that of A5 hydroxy group which involved in IHB in PIC, so the activity of A5-I is increased. To sum up, the O‒H BDE of PIC shows remarkable dependence on the pattern of IHB.

**Fig 12:**
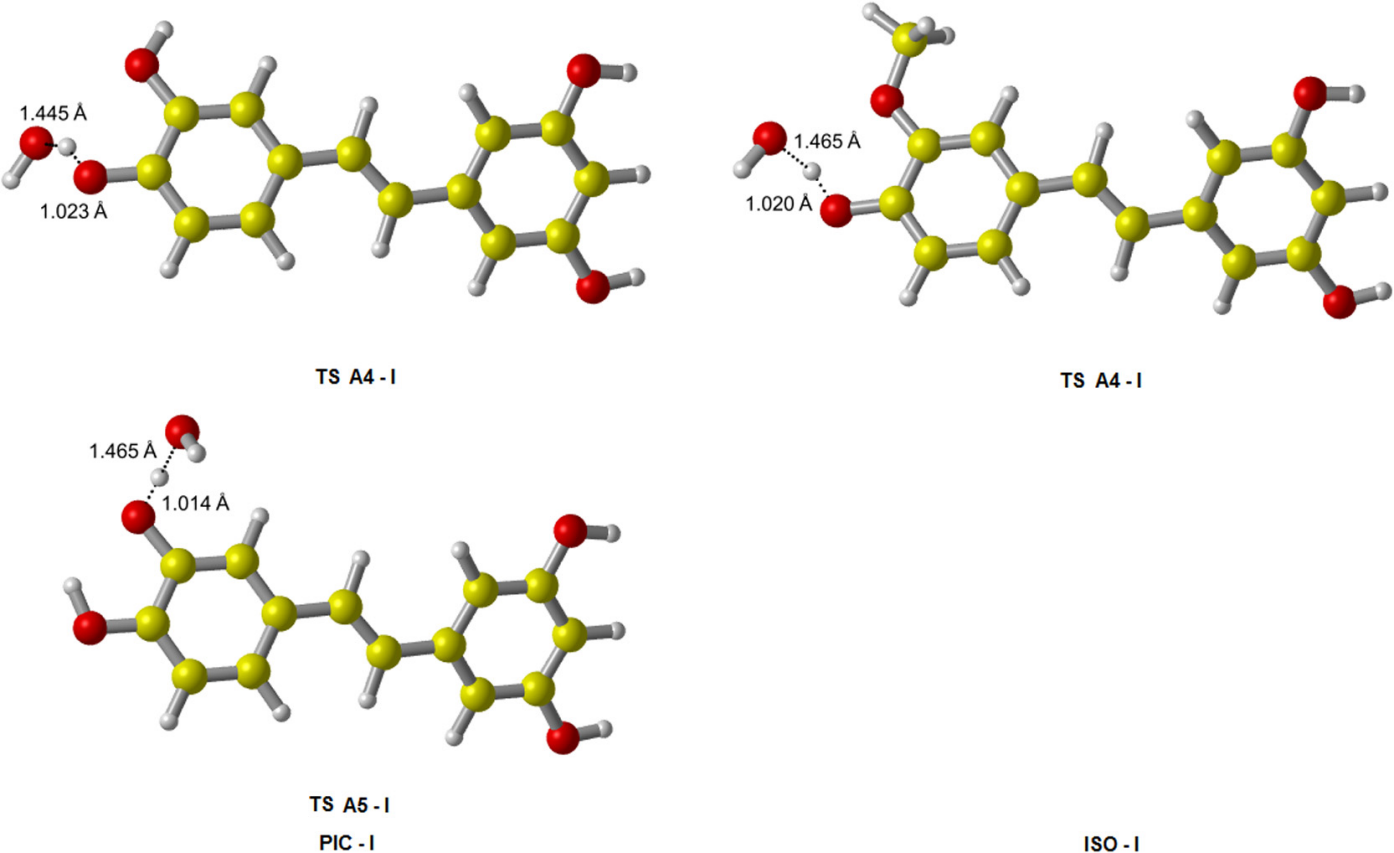
The transition state geometries of PIC-I and ISO-I from ABS reactions initiated by •OH. The elongations of the breaking bonds are smaller than those of the forming bonds, indicating that these TS are all reactant-like, i.e. these reactions are all exothermic in light of the Hammond’s postulate. These agree with our calculated results in Table 7.

Then we identified all TS of PIC-I and ISO-I scavenging •OH in the gas phase, their fully optimized geometries are shown in Fig 12. We also obtained the barrier heights of all pathways, along with the corresponding barrier heights of PIC and ISO, listed in Table 8. According to our previous discuss, the most active ABS site of PIC scavenging •OH is A4. But from this Table, it was interestingly found that the most active site of the PIC-I has changed: the barrier height of pathwayA5-I is lower than that of A4-I, A5-I becomes the easiest site abstracted by •OH. This can be explained that, for PIC-I, the H atom of A4-I hydroxy group which involved in IHB is more stable, therefore it needs more energy to be abstracted. And this is agreement with the change of its BDE. While for the free-OH group of A5-I, its product radical is stabilized by the IHB where adjacent A4-I OH interacts with its O atom. The presence of IHB decreased the O‒H BDE of A5-I, hence it becomes the most active-OH group of PIC-I with •OH both in the gas phase and water phase.

By comparison the Figs 1 and 11, we can see that there is no IHB in the structure of ISO. While for ISO-I, its H atom of A4-I group forms a IHB with the O atom of the adjacent methoxy group (A5-I). Table 8 shows that the difference of O‒H BDE of A4 and A4-I is minor. But the barrier height of pathway A4-I is especially higher than that of A4 in ISO. This proved that the presence of IHB could decreases the activity of the-OH group involved in the H-bond formation.

In conclusion, IHB has important effect to the activity of-OH group of PIC and ISO. The existence and pattern of IHB can potentially lead to completely different theoretically estimated for their H-transfer activity. Therefore the conformational effect is a important factor that should not be neglected in investigating antioxidant activity of PIC and ISO analogues.

## Conclusions

In this work, the antioxidation mechanisms behind •OH and •OOH scavenging activities of PIC and ISO were studied by using a quantum mechanical method at the M05-2X/6-311++G(d,p) level of theory. The reaction Gibbs energy, reaction enthalpy and reaction potential barrier height were computed in the gas phase and water. Two mechanisms, ABS and RAF, were investigated.

To scavenge •OH, both of ABS and RAF mechanisms are thermodynamically and kinetically feasible for PIC and ISO. For ABS mechanism, A4-hydroxyl group is the most active site of PIC and ISO both in the gas phase and water. The structure difference of A4/A'4 with other hydroxyl groups of PIC/ISO is that there is a substituent group on its ortho-position. So we conclude that introducing adjacent-OH and-OCH_3_ group would increase the radical scavenging activity of PIC and ISO. PIC is more effective than ISO in scavenging •OH, because its ortho-dyhydroxyl moiety allows to share the H atom by forming the IHB, so the corresponding product is more stable. For addition mechanism, the sites of β are always more active than α for both of PIC and ISO.

To scavenge •OOH, we find that ABS is the only thermodynamically feasible mechanism for PIC; while for ISO, both of ABS and RAF mechanisms are viable. In the gas phase, A4-hydroxyl group is still the most active site for PIC and ISO to scavenge •OOH by ABS mechanism, and the activity of PIC is more prominent than ISO. But in water, due to the solvent effect, A5-hydroxyl group becomes the most active site of PIC, at the same time PIC loses its superiority of scavenging •OOH in contrast to ISO. So the antioxidation activity of PIC is mainly controlled by the benzene ring A, and ISO is more effective than PIC in eliminating •OOH in organisms.

The changes of conformation have great influence on the antioxidant activity of PIC and ISO. The activity of the-OH group which participates in the formation of IHB will be decreased, while the activity of free-OH group will be increased.

Although both of PIC and ISO have a lower ability to scavenge •OOH than •OH, considering •OOH may also have a significant contribution to the oxidation in biological media, we conclude that PIC and ISO are very good antioxidants. To sum up, our study offers a deep understanding on the relationship of antioxidation mechanism with structure property of PIC and ISO.

## Supporting Information

S1 Fig The optimized geometries of the reactant complexes for the reactions of PIC with •OH in the gas phase. (TIF)

S2 Fig The optimized geometries of the reactant complexes for the reactions of ISO with •OH in the gas phase. (TIF)

S3 Fig The optimized geometries of the product complexes of PIC and ISO from ABS reactions initiated by •OOH in the gas phase. (TIF)
