# Supplementary figures and images for: Quantum Chemical Study on the Antioxidation Mechanism of Piceatannol and Isorhapontigenin toward Hydroxyl and Hydroperoxyl Radicals
Source: PLoS One. 2015 Jul 15;10(7):e0133259. doi: 10.1371/journal.pone.0133259 (PMC4503757; doi:10.1371/journal.pone.0133259)

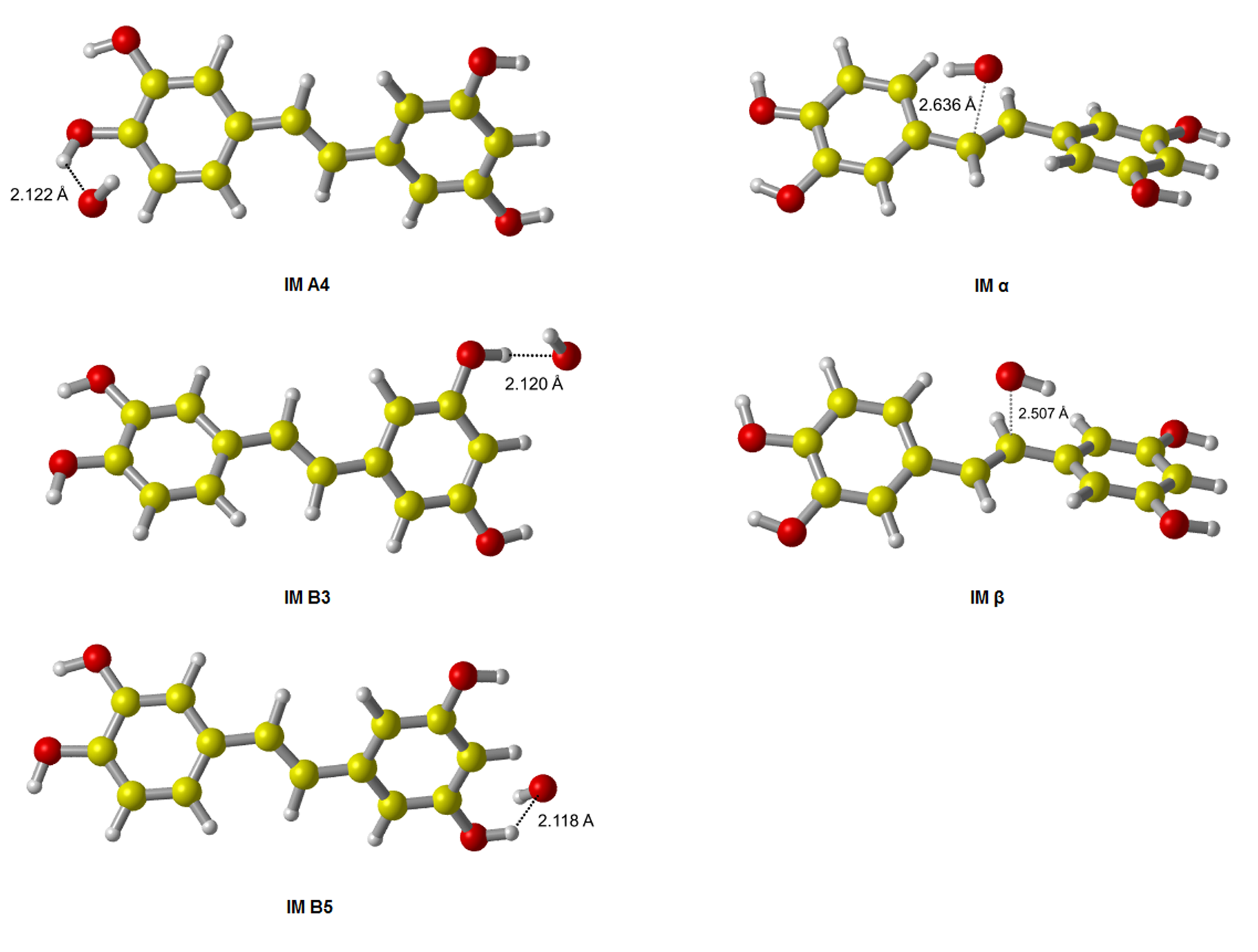

Supplement: S1 Fig — (TIF) [file pone.0133259.s001.tif]

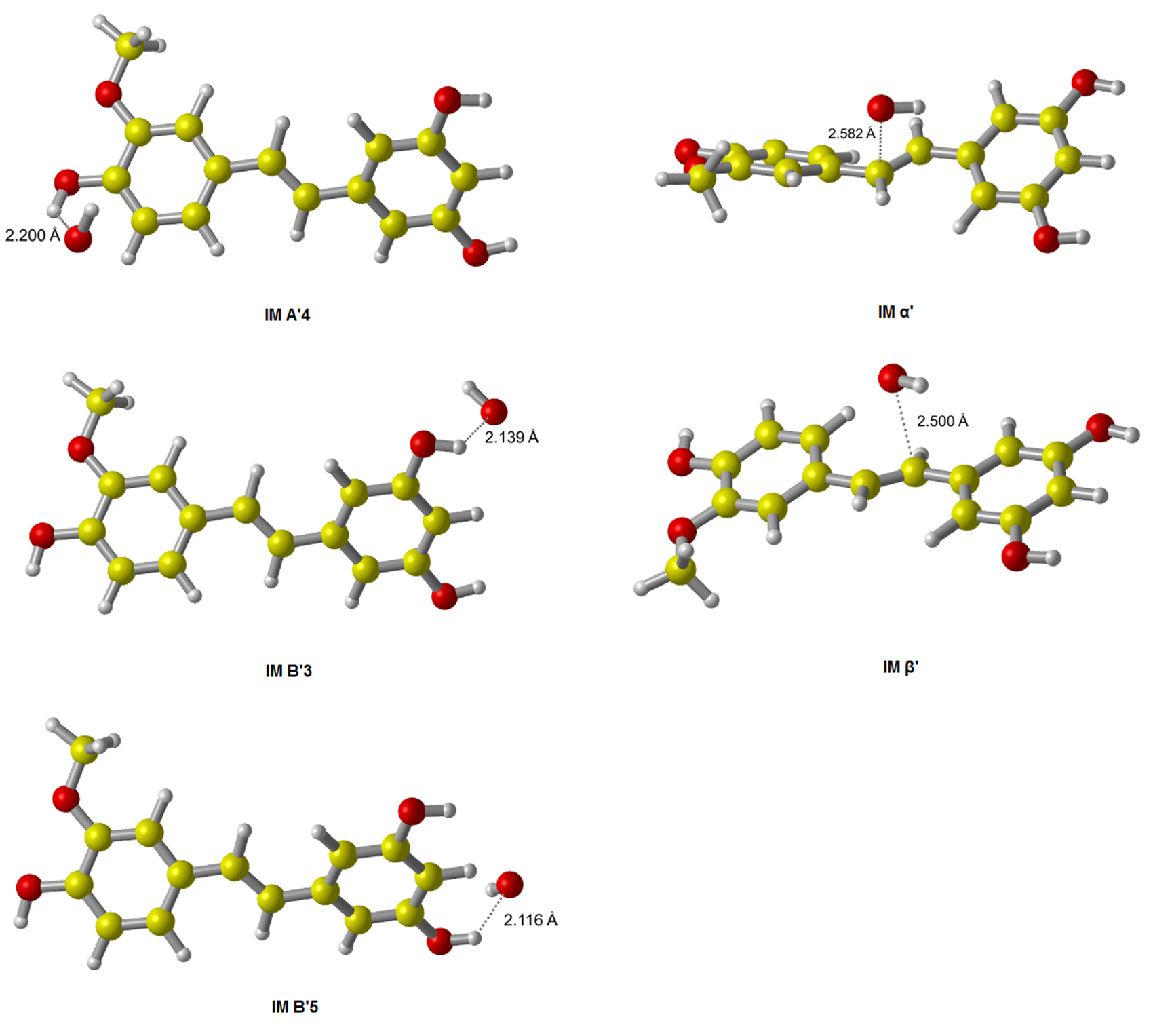

Supplement: S2 Fig — (TIF) [file pone.0133259.s002.tif]

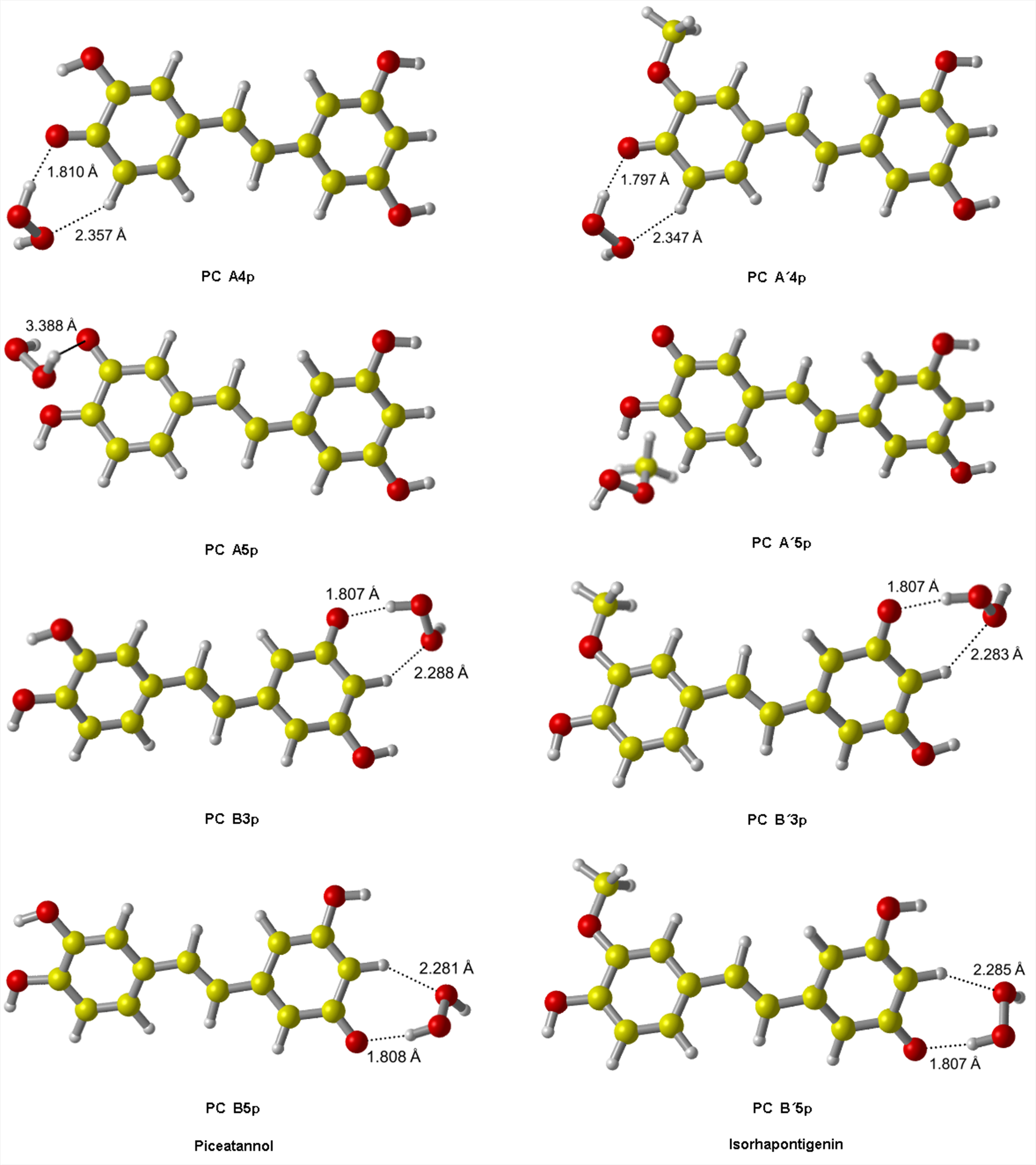

Supplement: S3 Fig — (TIF) [file pone.0133259.s003.tif]
